# Key Motivators and Framework for Integrated Care by Family Physician Team Members in Urban China

**DOI:** 10.34172/ijhpm.8781

**Published:** 2025-09-08

**Authors:** Weizhuo Chen, Zihui Xiong, Huiyan Fang, Wenqi Xiao, Ting Ye

**Affiliations:** ^1^School of Medicine and Health Management, Tongji Medical College, Huazhong University of Science and Technology, Wuhan, China.; ^2^Research Center for Rural Health Service, Key Research Institute of Humanities & Social Sciences of Hubei Provincial Department of Education, Wuhan, China.

**Keywords:** Integrated Care, Family Physician Team, Motivators, DEMATEL

## Abstract

**Background:**

This study aimed to develop an analytical framework to investigate the key motivators influencing family physician team members (FPTMs) in delivering integrated care at the primary healthcare (PHC) level within urban China. The objective was to identify critical factors based on cause and effect relationships, with the ultimate aim of enhancing the integration of primary care and public health services.

**Methods:**

A mixed-methods design was implemented, integrating thematic analysis and the Decision-Making Trial and Evaluation Laboratory (DEMATEL) method. Data were gathered from semi-structured interviews with 24 participants, comprising FPTMs and administrators from Wuhan, Shenzhen, and Shanghai, collected between January and July 2022. The thematic analysis facilitated the construction of an initial framework of motivators, while the DEMATEL method was employed to examine and map the interdependent relationships among these motivators.

**Results:**

The analysis distilled 64 first-order concepts into 15 second-order themes, which were then categorized into four broader dimensions: Government agencies, PHC institutions, family physician teams (FPTs), and residents. Motivators at the government level, such as resource allocation and the development of information technology (IT) infrastructure, were identified as proactive forces driving change. In contrast, resident-level factors, including trust and adherence, were more reactive, shaped by external conditions and responsive rather than initiators of change.

**Conclusion:**

The findings emphasize the pivotal role of government leadership in fostering the adoption of integrated care. Key strategies include enhancing interdisciplinary team collaboration, optimizing performance evaluations, and refining incentive structures to boost FPTMs’ motivation. Equally important is the need to encourage residents to engage in proactive health management, promoting a collaborative care model that integrates both FPTMs and the communities they serve.

## Background

**Key Messages**

**Implications for policy makers**

- This study provides a clearer understanding of stakeholder responsibilities within integrated care policies and offers strategic recommendations for aligning incentives to harmonize the interests and objectives of all involved parties.
- In the early stages of promoting integrated care, governmental leadership could be leveraged to set clear guidelines and integrate primary care with public health information systems.
- For healthcare providers, it is crucial to strengthen the organization and management of primary healthcare (PHC) institutions related to FPTs, enhance interdisciplinary team collaboration, performance evaluations and incentive structures to boost family physician team members’ (FPTMs’) intrinsic motivation.

**Implications for the public**

This study highlights the importance of aligning integrated care delivery with the actual needs and perceptions of the population. In the context of an aging society and the rising prevalence of chronic conditions, guiding residents to adopt healthier perceptions and behaviors is essential. The findings offer practical strategies to improve service responsiveness and strengthen resident engagement. Enhancing motivators for family physician team members (FPTMs) may improve the accessibility and quality of integrated care, build trust between residents and providers, and ultimately support healthier lifestyles and greater self-management. These improvements are critical to meeting population health needs more effectively and advancing the long-term goals of integrated primary care.

Globally, the shift from infectious diseases to chronic conditions and the rise of aging populations are pushing health systems towards greater integration.^1,2^ In 2015, the World Health Organization’s (WHO’s) “Global Strategy on People-Centered and Integrated Health Services” promoted a prevention-first health service model, underscoring the critical role of primary healthcare (PHC).^3^ From a PHC perspective, promoting the integration of primary care and public health can effectively contribute to improved population health outcomes.^4-6^

 Nations worldwide are striving to bridge the gap between primary care and public health. Examples include the Patient-Centered Medical Home in the United States,^7^ the community-oriented primary care initiatives in countries such as South Africa, Israel, and Cuba,^8,9^ and the diagnoses treatment combination in the Netherlands, based on People-Centered Integrated Care, which has further developed into diabetes care groups.^10^ Integrated care in China involves comprehensive and continuous services throughout the life course, including health promotion, disease prevention, diagnosis, treatment, disease management, rehabilitation, and hospice care.^11^ China has proposed delivering integrated care through collaborative, interdisciplinary family physician teams (FPTs) in 2018. FPTs consist of an interdisciplinary team that must include at least one general practitioner and one nurse, with the potential to also include public health physicians, pharmacists, health management specialists, rehabilitation therapists, social workers, and other healthcare professionals.^12^ This model mirrors Canada’s family health teams and similar collaborative team-based models in the UK, the US, Cuba, and other countries.^13-16^

 However, many primary care facilities in China still lack the capacity or motivation to provide high-quality preventive and public health services, despite strong governmental emphasis on chronic disease prevention.^17,18^ Moreover, in some regions, especially those with limited health workforce, primary care providers are gradually moving away from their main clinical duties and placing more focus on preventive care than on treatment. Both situations together have contributed to the ongoing divide between clinical care and public health at the PHC level in China.^19,20^

 Previous research on integrated care at the PHC level has primarily focused on integrated care delivery systems,^21^ as well as the efficiency and effectiveness of these services.^22,23^ Globally, efforts are underway to explore effective approaches for integrating PHC and public health, including modifying payment methods, reforming compensation systems, and employing team-based approaches.^13,24^ Motivators play a crucial role in stimulating the intrinsic motivation of healthcare professionals and shaping their service delivery behaviors. The inadequacy of such incentives, however, hampers the provision of integrated care from PHC level.

 Extensive research exists on incentivizing healthcare professionals, with motivators typically categorized into monetary and non-monetary types. Monetary motivators include salaries, fee-for-service, capitation, and mixed payment methods.^25-28^ Non-monetary motivators include quality improvement, a culture of learning, professional reputation, transparency, service coordination, and information technology (IT).^26,28^ Some scholars argue that “Family physicians with adequate financial and physical resources function most effectively in interdisciplinary teams, providing comprehensive health services globally.”^29^ While both monetary and non-monetary motivators are important, it remains unclear whether they fully meet the expectations of family physicians. Research indicates that while economic incentives positively impact short-term performance, once material needs are met, individuals shift toward higher-level needs such as relationships and personal growth. Consequently, economic incentives may negatively impact long-term performance, leading to motivational imbalances.^30^ Thus, a greater focus on non-monetary motivators and comprehensive research on various forms of motivation is necessary.

 Currently, incentive challenges within China’s FPTs are impeding the full utilization of family physician team members (FPTMs) in delivering integrated care services. Factors such as low remuneration, insufficient collaboration between community health services and hospital-based specialty services, limited professional skills, restricted career development opportunities, and a lack of trust from residents contribute to low work motivation among FPTMs.^31,32^ Research suggests that providing integrated care can better satisfy FPTMs’ needs for relationships, foster strong doctor-patient relationships, and enhance their sense of purpose and professionalism.^33-35^ A literature review highlights the urgent need to motivate FPTMs to engage in integrated care. This requires a systematic understanding of the various motivators to better mobilize and sustain their enthusiasm for integrated care.

 Therefore, this study aims to develop an analytical framework to examine the motivators for FPTMs within the context of integrated care and to analyze their interrelationships. The objective is to identify key factors based on cause and effect relationships and the extent of their interdependence, providing a clearer understanding of how to advance the integration of primary care and public health at the PHC level.

## Methods

 Given the complexity of integrated care work within FPTs and the diverse motivators influencing FPTMs, we initially conducted semi-structured, in-depth individual interviews with family physicians and administrators at primary care institutions. Through thematic analysis, we identified the core needs of FPTMs in the context of integrated care, which enabled the construction of an analytical framework for motivators. Subsequently, we applied the Decision-Making Trial and Evaluation Laboratory (DEMATEL) method to analyze the cause and effect relationships among factors, identifying cause group and effect group factors to determine key motivators for FPTMs in delivering integrated care services. Data was collected between January 1, 2022, and July 1, 2022, and all participants provided informed consent.

###  Thematic Analysis Method

####  Sample and Recruitment

 To ensure this study was grounded in the specific needs of FPTMs, a purposive sampling method was employed. The participants were primarily key implementers of integrated care policies at the PHC level, including both administrators and FPTMs. The sampling process followed to the principle of maximum variation,^36,37^ considering factors such as demographic characteristics, years of service, and roles within the teams to enable data triangulation. The sample size was determined according to the principle of theoretical saturation, resulting in 24 participants: Six administrators and 18 FPTMs from Wuhan, Shenzhen, and Shanghai. Notably, four of the six administrators also served as FPTMs.

####  Data Collection

 This study employed semi-structured interviews to collect data. The guide questions were designed based on literature review and observational insights, with a focus on motivators for delivering integrated care at the PHC level. The interviews explored participants’ perspectives on integrated care, existing support and security measures, key factors for effective service delivery, and motivating elements for service provision. Interviews were conducted both in person and online, with each session lasting between 10 and 40 minutes. Follow-up questions were asked in response to participants’ answers, aligning with the guide questions. After developing an initial conceptual framework, follow-up interviews were conducted with key individuals to confirm and refine the core themes and concepts.

####  Data Analysis

 This study employed thematic analysis of qualitative data in accordance with the recommendations of Gioia et al,^38^ using an inductive approach that allows for negotiation between data and theory. Coding was conducted using MAXQDA software, with an experienced researcher serving as the primary coder and additional researchers acting as “devil’s advocates.”^39^ This iterative process enhanced the accuracy and consistency of the codes through continuous questioning and the introduction of alternative interpretations.

 This study was grounded in the analytical framework of “1st-order concepts, 2nd-order themes, and aggregate dimensions” for constructing data structure.^38^ Three data analysis steps were undertaken.

 Step 1. The participants’ original expressions were distilled into descriptive phrases, forming first-order concepts—distinct, informant-centric labels that directly reflect participants’ own words and perceptions regarding integrated care at the PHC level.^40^ As the number of first-order concepts increased, similarities and differences among them were examined to reduce redundancy and identify meaningful patterns for further abstraction.

 Step 2. First-order concepts were compared and grouped, and similar concepts were abstracted into second-order themes, which were typically theory centric or researcher centric. These themes represent the researchers’ interpretive lens applied to participants’ views on integrated care motivators. As this analysis progressed, the researcher gained a deeper understanding of integrated care motivators and the relevant literature. We then focused on the emerging concepts and, once theoretical saturation was reached, conducted further analysis to distill 2nd-order themes into ‘‘aggregate dimensions.”^40^ Aggregate dimensions are higher-order, overarching theoretical constructs that capture the core domains of meaning emerging from the data. This study integrated previously research and drew upon Franco and colleagues’ conceptual framework for health personnel incentives,^41^ developed from the perspective of health administration reform,^42^ to summarize aggregate dimensions.

 Step 3. A model was constructed to analyze the dynamic interrelationships between second-order themes and aggregate dimensions, illustrating how the interests of various stakeholders directly or indirectly motivate FPTMs to deliver integrated care.

 Throughout the analysis, we cycled between data, concepts, themes, dimensions, and relevant literature to help delineate the boundaries between each concept and the derived themes, advancing the process of conceptualization.^38^

###  DEMATEL Method

 The DEMATEL method, developed by the Battelle Institute in Geneva in 1972, was designed to explore and address complex, interrelated problems. As a structural modeling methodology, DEMATEL does not require the assumption of independence among elements. By constructing matrices or directed graphs based on expert judgments, the method visualizes complex interdependent relationships, referring specifically to the perceived directional influence between factors. DEMATEL quantitatively illustrates both the connections and strengths among these elements, thereby identifying critical factors within intricate networks.^43^ Due to its effectiveness in clarifying complex relationships, the method has been widely applied in various domains, including healthcare decision-making and technology utilization.^44,45^

####  Data Collection

 This study quantified the influence between motivators related to integrated care within FPTs by gathering expert opinions. To ensure scientific rigor and comprehensiveness, questionnaires were distributed to 20 experts, comprising 7 university scholars, 5 health administration personnel, 3 PHC administrators, and 5 FPTMs. The experts received an email containing a link to an online questionnaire. The questionnaire included an introduction, definitions of the motivators relevant to integrated care among FPTMs, and a scoring table for assessing the correlations between these factors. Experts evaluated the extent to which each factor influenced another based on theoretical knowledge, professional experience, or peer understanding. Specifically, they assessed whether the relationship between factors was causal, assigning an influence score ranging from 0 (no influence) to 3 (very strong influence).

####  Data Analysis

 Based on expert ratings, this study used the DEMATEL method to visualize the complex interdependent relationship structure among motivators for FPTMs. By constructing matrices, the analysis clarified the cause and effect relationships and the strength of influence between various motivators, identifying key motivators within a network of interrelated incentives. Following the principles of the DEMATEL algorithm, this study adhered to four steps to identify key motivational factors^43,44,46^:

 Step 1. Compilation of the direct-relation matrix *A*. The direct influence between any two factors is evaluated by each expert using an integer score of 0, 1, 2, and 3, representing the extent to which factor *F*_i_ influences factor *F*_j_. For *i*= *j*, the diagonal elements are set to zero, which indicates no influence. The scoring results from each expert can be gained as a direct-relation matrix that is an *n × n* and non-negative answer matrix $A_{k}={\left[a_{ij}^{k}\right]}_{n\times n}$ , *a*_ij_ represents the numerical score assigned by experts to this influence. To aggregate all opinions from experts, the average matrix $A_{k}={\left[a_{ij}^{k}\right]}_{n\times n}$ can be constructed as follows:


(1) $$a_{ij}=\frac{1}{m}\sum_{k=1}^{m}a_{ij}^{k},\;\;i,j=1,2,...,n.$$

Step 2. Normalization of the direct-relation matrix *A*. The normalized initial direct-relation matrix *X* is calculated by using the following equation:


(2) $$X=\frac{A}{\;max_{\;1\leq j\leq n\sum_{j=1}^{n}a_{ij}}},\;\;0\leq x_{ij}\leq 1$$

Step 3. Calculation of the total-relation matrix *T*. The total-relation matrix captures the overall influence that each factor exerts on others, including both direct and indirect effects transmitted through intermediate factors. The total-relation matrix can be calculated using the following equation:


(3) $$T=X{\left(I-X\right)}^{-1},\;\;I=\text{identity}\;\text{matrix}.$$

Step 4. Calculation of prominence and relation values and drawing of causal network diagram. The vectors *R* and *C*, representing the sum of the rows and the sum of the columns from the total-influence matrix *T*, are defined by the following equation:


(4) $$R={\left[r_{i}\right]}_{n\times 1}={\left[\sum_{j=1}^{n}t_{ij}\right]}_{n\times 1}$$

*r*_i_ is the *i*th row sum in the matrix *T* and represents the sum of effects dispatching from factor *F*_i_ to the other factors. Similarly, *cj* is the *j*th column sum in the matrix *T* and depicts the sum of effects that factor *F*_j_ is receiving from the other factors. The sum *R + C* referred to as “Prominence,” illustrates the overall influence a factor has within the system, indicating the degree of centrality it holds. Conversely, *R – C* termed “Relation,” shows the effect a factor contributes. A positive *R – C* value classifies the factor into the cause group, while a negative value indicates that it belongs to the effect group. This study utilized MATLAB to perform the calculations described above, focusing on *R + C* while integrating *R – C*, *R*, and *C* to identify key motivators.

## Results

###  Thematic Analysis

**Figure 1:**
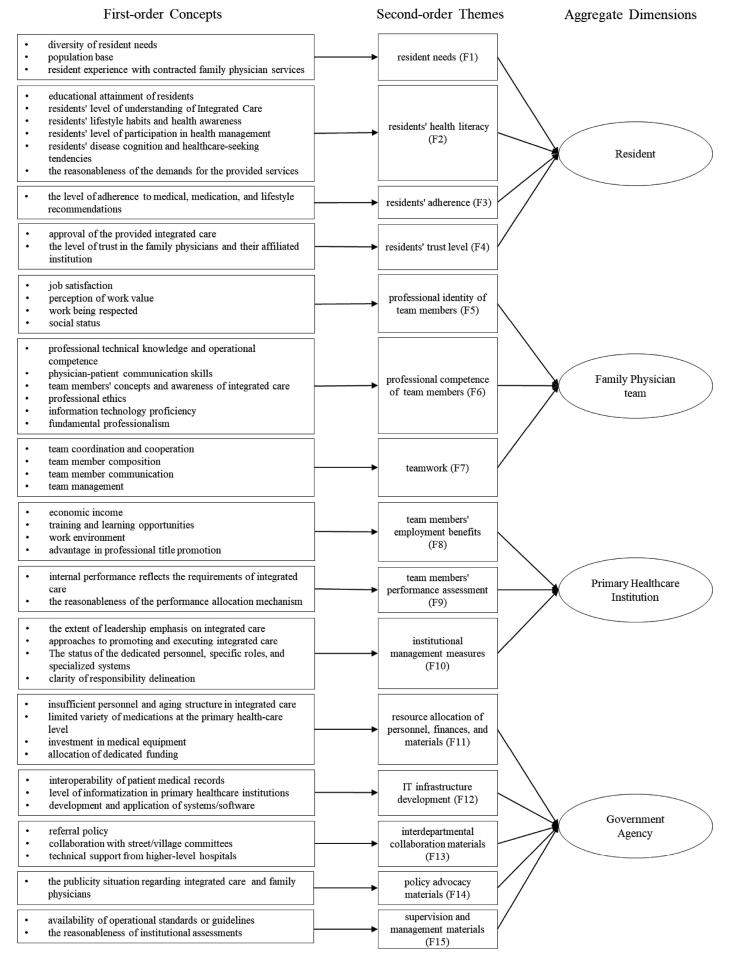
Data Structure. Note: Motivators were identified from qualitative data and assigned codes F1 to F15 according to their emergence in the thematic coding process. This order is maintained throughout figures and analysis for consistency.

 This study constructed a data structure through thematic analysis, as illustrated in Figure 1. First-order concepts, directly relevant to participants, led to the development of second-order themes, which were further refined into aggregate dimensions, forming the motivator framework. The study identified 64 first-order concepts. From these, Based on these, and in consideration of real-life scenarios where FPTMs provided integrated care, 15 second-order themes were abstracted. Through repeated comparison and analysis, these themes were distilled four aggregate dimensions: Resident, FPT, PHC institution, and government agency. Together, these four entities formed an organic incentive system that directly or indirectly motivates FPTMs to deliver integrated care.

**Figure 2:**
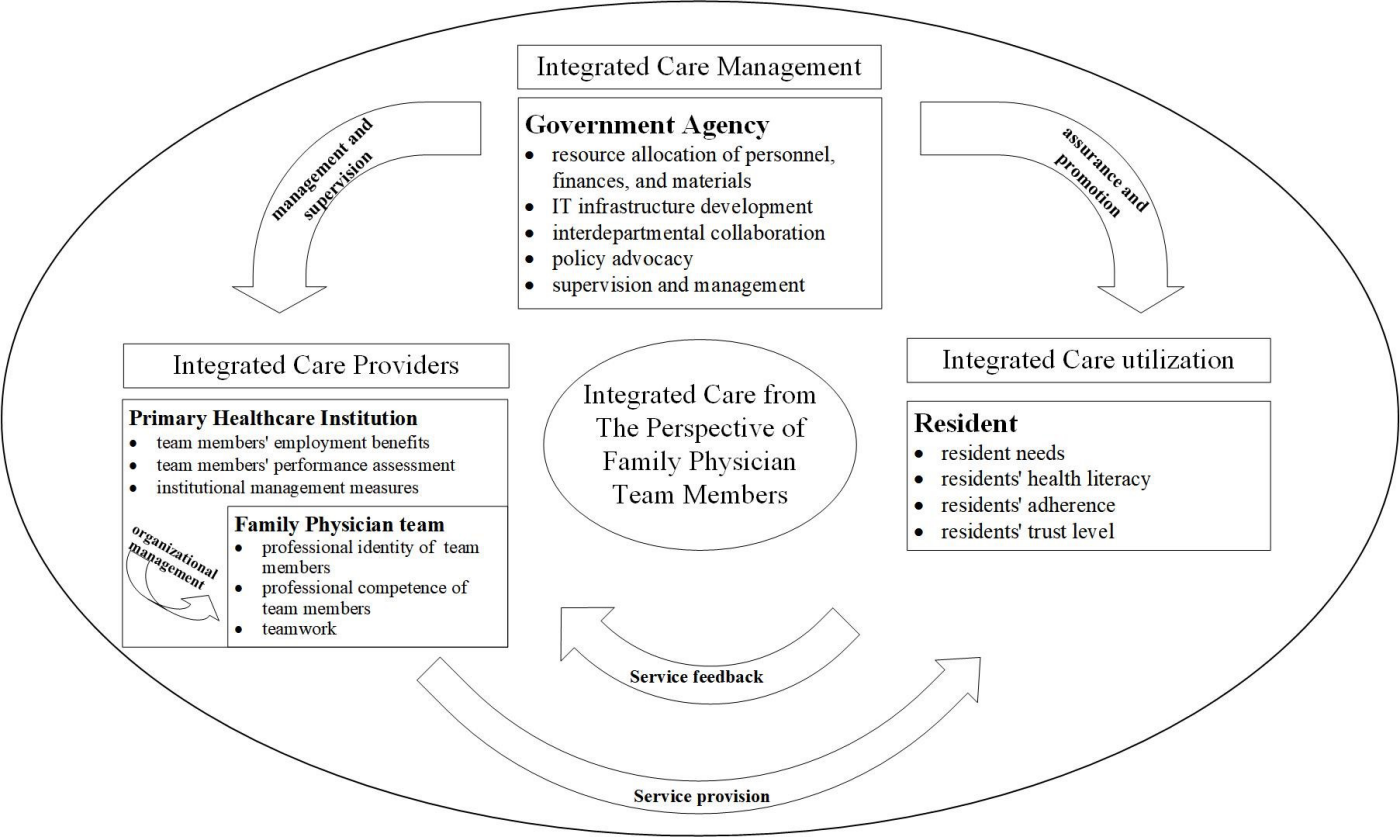
Analytical Framework for Motivators.

 This study developed a dynamic motivator model (Figure 2), illustrating how residents, FPTs, PHC institutions, and government agencies incentivized FPTMs to provide integrated care. Personal factors within FPTMs acted as critical internal drivers of direct motivation.

 “*For me, professional identity and the social status associated with my profession bring a greater sense of fulfillment than financial rewards” *[P2].

 “*Healthcare providers must continually improve their professional skills—if your skills are lacking, patients simply will not come. At the same time, providers also need to shift their mindset. You have to truly value the integrated care and prevention; if you don’t, it won’t work, no matter what” *[P6].

 At the institutional level, management mechanisms of PHC institutions served as significant internal environmental factors, motivating the delivery of integrated care.

 “*Efforts in integrated care and prevention are hardly reflected in performance evaluations. Even the most proactive physicians receive the same compensation regardless of the additional work they put in”* [P8].

 “*The division of responsibilities is unclear. I am essentially taking on two roles at once, and both carry a heavy workload”* [P13].

 External environmental factors at the resident and government levels were also crucial. As users of integrated care, residents provided feedback that directly influenced enthusiasm for service provision.

 “*At the very least, all residents need to have an awareness of disease prevention. Without that awareness, no matter how much we try to communicate, it’s ineffective” *[P3].

 “*Patient trust, in my view, serves as a stronger motivator on a psychological level than any material incentive”* [P15].

 Government agencies directly motivated FPTMs by advancing informatization, offering technical support, and establishing operational guidelines, while also indirectly fostering motivation by incentivizing PHC institutions and residents.

 “*I hope that all performance indicators can be fully digitized and automated, so that we no longer need to manually search through records every day. Information systems should be able to generate outputs automatically. It is essential to free primary care providers from being tied to their computers, allowing them to devote more time to communicating with and serving patients”* [P4].

 “*Higher-level authorities may have certain expectations for the integrated care and set performance targets for the grassroots level, but in practice, implementation is extremely difficult”* [P14].

###  DEMATEL Model Construction

 After averaging the scores of 20 experts, the direct-relation matrix *A* for the motivators of the integrated care among FPTMs was obtained (Table 1).

**Table 1:** Direct-Relation Matrix^a^

**Motivators**	**F1**	**F2**	**F3**	**F4**	**F5**	**F6**	**F7**	**F8**	**F9**	**F10**	**F11**	**F12**	**F13**	**F14**	**F15**
F1	0.00	2.26	2.37	2.32	2.42	2.11	1.79	1.89	2.11	1.84	2.11	1.89	1.95	2.16	1.89
F2	2.53	0.00	2.68	2.63	2.42	1.84	1.74	1.74	1.74	1.84	1.58	1.53	1.58	1.84	1.47
F3	2.42	2.21	0.00	2.53	2.68	2.05	2.05	2.26	2.05	1.89	1.95	1.63	1.79	1.89	1.74
F4	2.53	2.21	2.68	0.00	2.79	2.26	2.00	1.95	2.00	1.89	1.63	1.58	1.47	1.95	1.68
F5	2.16	2.11	2.32	2.53	0.00	2.84	2.63	2.47	2.26	2.11	1.89	1.68	1.89	1.89	1.89
F6	2.84	2.53	2.68	2.84	2.74	0.00	2.74	2.42	2.26	2.26	1.89	2.05	2.11	2.05	2.05
F7	2.84	2.32	2.74	2.68	2.42	2.42	0.00	2.32	2.37	2.26	1.95	1.95	1.95	1.95	1.95
F8	2.11	1.89	1.95	1.95	2.89	2.58	2.58	0.00	2.47	2.37	2.26	1.95	1.95	1.89	2.11
F9	2.16	2.05	2.32	2.00	2.42	2.32	2.68	2.79	0.00	2.37	2.32	2.05	1.89	1.79	2.16
F10	2.21	2.05	2.21	2.42	2.47	2.37	2.68	2.63	2.74	0.00	2.68	2.63	2.68	2.37	2.63
F11	2.42	2.00	2.21	2.16	2.53	2.42	2.53	2.84	2.58	2.47	0.00	2.63	2.53	2.32	2.42
F12	2.16	2.11	2.05	2.16	1.84	2.05	2.37	2.11	2.32	2.37	2.37	0.00	2.58	2.21	2.58
F13	2.42	2.05	2.32	2.42	2.32	2.32	2.63	2.32	2.32	2.26	2.37	2.26	0.00	2.05	2.32
F14	2.47	2.26	2.32	2.53	2.53	2.21	1.95	1.68	1.84	2.21	2.21	2.05	2.21	0.00	2.11
F15	1.95	1.68	2.00	2.26	2.11	2.21	2.47	2.26	2.63	2.79	2.37	2.37	2.26	2.05	0.00

^a^See Figure 1 for definitions of F1–F15.

 The direct impact matrix *A* changed into the total-relation matrix *T* using equation (2) and (3) (Table 2).

**Table 2:** Direct-Relation Matrix (T)^a^

**Motivators**	**F1**	**F2**	**F3**	**F4**	**F5**	**F6**	**F7**	**F8**	**F9**	**F10**	**F11**	**F12**	**F13**	**F14**	**F15**
F1	0.51	0.52	0.57	0.58	0.60	0.55	0.55	0.54	0.54	0.52	0.51	0.49	0.50	0.50	0.50
F2	0.54	0.43	0.54	0.55	0.56	0.51	0.52	0.50	0.50	0.49	0.47	0.45	0.46	0.46	0.46
F3	0.57	0.52	0.51	0.58	0.60	0.55	0.56	0.55	0.54	0.53	0.51	0.48	0.49	0.49	0.49
F4	0.57	0.51	0.57	0.50	0.60	0.54	0.55	0.53	0.53	0.52	0.49	0.47	0.48	0.48	0.48
F5	0.60	0.54	0.59	0.61	0.56	0.59	0.60	0.58	0.57	0.56	0.53	0.50	0.52	0.51	0.52
F6	0.66	0.59	0.65	0.66	0.68	0.56	0.65	0.62	0.61	0.60	0.57	0.55	0.56	0.55	0.56
F7	0.63	0.57	0.63	0.63	0.65	0.60	0.55	0.60	0.60	0.58	0.55	0.53	0.54	0.53	0.54
F8	0.60	0.54	0.59	0.60	0.64	0.59	0.61	0.52	0.59	0.57	0.55	0.52	0.53	0.52	0.53
F9	0.61	0.55	0.61	0.61	0.64	0.59	0.61	0.60	0.52	0.57	0.55	0.52	0.53	0.52	0.54
F10	0.67	0.60	0.66	0.67	0.70	0.65	0.67	0.65	0.65	0.56	0.61	0.59	0.60	0.59	0.60
F11	0.66	0.59	0.65	0.66	0.69	0.64	0.65	0.64	0.64	0.62	0.53	0.58	0.58	0.57	0.59
F12	0.61	0.55	0.60	0.61	0.62	0.59	0.61	0.58	0.59	0.57	0.55	0.47	0.55	0.53	0.55
F13	0.63	0.57	0.62	0.63	0.65	0.61	0.63	0.60	0.60	0.59	0.57	0.54	0.49	0.54	0.56
F14	0.60	0.54	0.59	0.61	0.62	0.58	0.58	0.56	0.56	0.56	0.54	0.51	0.52	0.46	0.52
F15	0.60	0.54	0.60	0.62	0.63	0.59	0.61	0.59	0.60	0.59	0.56	0.54	0.54	0.53	0.48

^a^See Figure 1 for definitions of F1–F15.

 It was necessary to set up a threshold value to filter out some negligible effects in practice and show the valuable information. The average of the elements in matrix *T* was computed to determine the threshold value in this study. The average of the elements in matrix *T* was 0.566.

 According to the total impact relation matrix *T*, the sum of rows (*R*) and the sum of columns (*C*) of factors in the matrix are calculated, as shown in Table 3, with their prominence (*R + C*) and relation (*R – C*) being obtained.

**Table 3:** Prominence and Relation^a^

	**R**	**C**	**R+C**	**R-C**	**Group**
F1	7.96	9.05	17.00	-1.09	Effect
F2	7.42	8.16	15.58	-0.73	Effect
F3	7.96	8.96	16.92	-1.01	Effect
F4	7.81	9.10	16.92	-1.29	Effect
F5	8.38	9.41	17.79	-1.03	Effect
F6	9.06	8.75	17.8	0.31	Cause
F7	8.72	8.94	17.66	-0.21	Effect
F8	8.48	8.65	17.14	-0.17	Effect
F9	8.57	8.64	17.21	-0.07	Effect
F10	9.46	8.42	17.88	1.04	Cause
F11	9.27	8.07	17.35	1.20	Cause
F12	8.56	7.72	16.28	0.84	Cause
F13	8.83	7.87	16.70	0.96	Cause
F14	8.34	7.79	16.13	0.56	Cause
F15	8.62	7.92	16.54	0.70	Cause

^a^See Figure 1 for definitions of F1–F15.

 This study focused on *R + C* by combining *R – C*, *R*, and *C* to identify key motivators. Institutional management measures (F10), professional competence of team members (F6), and resource allocation of personnel, finances, and materials (F11) exhibited high prominence and were classified into the cause group, exerting strong influence over other motivators. These factors were critical for integrating medical and preventive services among FPTMs. Conversely, professional identity of team members (F5), resident needs (F1), residents’ adherence (F3), and residents’ trust level (F4) had low R but high C values, indicating they were classified into the effect group, easily influenced by cause group factors and closely linked to other factors, making them key motivators. Teamwork (F7) ranked fourth in prominence, both influencing and being influenced by other factors, establishing it as a key motivator. Although interdepartmental collaboration (F13), supervision and management (F15), and IT infrastructure development (F12) ranked lower in prominence, their classification as cause group factors with high relation values, indicates that they actively influence other factors within the system, rendering them essential motivators as well.

## Discussion

 This study, conducted in the context of integrated care at the PHC level in China, aimed to identify the motivators for urban community FPTMs. Integrated care is defined by its patient-centered orientation, focusing on organizing healthcare services around individuals’ needs rather than simply linking different components of the healthcare system and external services.^47^ Building on this understanding, we developed an analytical framework of motivational factors to determine the core drivers shaping integrated care implementation. Three key findings emerged: (1) From the perspective of integrated care, the motivators for FPTMs consist of 15 factors across four levels: Government agencies, PHC institutions, FPTs, and residents. These motivators were interrelated and exhibited motivational relationships across the levels. (2) Government-level motivators primarily are cause group factors, with the government playing a proactive role in significantly influencing other levels. These motivators were relatively stable and controllable. Key causal motivators included resource allocation of personnel, finances, and materials (F11), IT infrastructure development (F12), interdepartmental collaboration (F13), and supervision and management (F15). Additionally, professional competence of team members (F6) and institutional management measures (F10) were also key cause group motivators, with resource allocation of personnel, finances, and materials (F11) being the most proactive. (3) Resident-level motivators acted mainly as effect group factors, suggesting that residents were in a passive position, easily influenced by other factors. Key effect motivators included resident needs (F1), residents’ adherence (F3), residents’ trust level (F4), professional identity of team members (F5), and teamwork (F7), with residents’ trust level (F4) being the most susceptible to influence.

 This study categorized motivators based on the management, provision, and utilization of integrated care at the PHC level, constructing an analytical framework. Previous researches show that family doctors’ understanding of their work and income can enhance job stability and improve contracted services performance.^48^ Similar to Belgium’s experience with integrated care, the motivation for FPTMs to implement integrated care in China is also complex due to the involvement of multiple stakeholders.^49,50^ This study aligned with Franco and colleagues’ conceptual framework from the perspective of health sector reform.^42^ However, unlike Franco et al, who considered cultural and community influences, this study focused on urban community health service centers in China, where cultural uniformity excludes this dimension from thematic analysis. This study emphasized resident-level motivators, underscoring the close relationship between FPTs and residents under integrated care policies. PHC institutions and residents should theoretically provide feedback to the government. Institutions are expected to report service information and updates,^51^ while resident satisfaction should influence governmental supervision and evaluation of healthcare institutions and personnel.^52,53^ However, as this study focused on the positive motivators for FPTMs in delivering integrated care, these feedback mechanisms were not incorporated into the data structure.

 Among the causal key motivators, four out of five government-level motivators were deemed essential. The development of integrated care at the PHC level in China heavily depends on government support, as the government formulates integrated care policies.^54^ Primary care and public health are coordinated across various professional, institutional, and sectoral boundaries at the micro level.^4^ The government plays a comprehensive and coordinating role in micro level integration, facilitating the active participation of PHC institutions, FPTMs, and residents through resource allocation and support measures.^55^ This significantly impacts the FPTM motivation, making government-level motivators more proactive and stable compared to other levels. In the early stages of promoting integrated care, these motivators should be prioritized. Institutional management measures (F10) were key cause group motivators at the PHC institution level. Currently, FPTMs in China mainly consist of general practitioners, nurses, and public health physicians affiliated with PHC institutions. Integrated care reforms increase workload and pressure, posing new challenges for healthcare personnel.^5,56^ Additionally, an inadequate pay-for-performance system may undermine FPTM motivation to implement integrated care.^57^ The professional competence of team members (F6) was a key cause group motivator at the FPT level. Studies showed 41.75% of PHC personnel self-assessed their theoretical knowledge and professional capabilities as limited to their current roles.^58^ This study also found that FPTMs felt underqualified to provide integrated care, unable to meet residents’ needs, and sought to improve their skills for career advancement.

 Among the key effect motivators, three out of four at the resident level were influenced by cause group factors. This imbalance stems from disparities in power and influence, the government and healthcare providers typically possess more authority, information, and expertise compared to residents.^59^ Promoting good living habits, rational medical views, and a proper understanding of integrated care and FPTs among residents require guidance from the government and healthcare providers.^60^ Insufficient health literacy among residents can result in suboptimal outcomes for integrated care services, such as chronic disease management.^61^ Conversely, resident feedback can drive improvements in integrated care.^62^ The DEMATEL method validated the constructed motivational framework, highlighting the proactive role of the government and the passive role of residents. Studies indicate residents in China demonstrate low willingness to sign up with FPTs, partly due to a lack of trust in their clinical capabilities,^63^ which hinders compliance with chronic disease management. Additionally, some residents misunderstand the role of FPTs, expecting them to function as “private doctors,” thereby increasing the communication burden on team members.^32^ Professional identity of team members (F5) and teamwork (F7) were key effect motivators at the FPT level. Effective collaboration within FPTs enhances integrated care. Comprehensive integrated care requires significant effort from FPTMs in their daily work. In the long term, a multidisciplinary team approach ensures better service coordination and continuity.^64^ For instance, while general practitioners provide clinical services, nurses or public health physicians should assist with preventive services, such as health education and management to maximize integrated care benefits of integrated care.^65^ Additionally, a study in Jiangsu Province confirmed that professional identity influences work cognition, competency, and stability among family physicians.^31^ Therefore, enhancing professional identity can motivate FPTMs to actively engage in integrated care.

 Several limitations should be acknowledged. First, expert scoring might be influenced by variations in decision-making contexts. Although mean-based aggregation is considered relatively optimal, it may still yield counterintuitive outcomes, limiting its full capture of real-world complexity.^66^ Second, our study uses the concept of causality as defined by the DEMATEL method, where it refers to expert-perceived directional influences between factors. This form of causality is structural and interpretive rather than based on empirical testing. Future research could help validate these patterns through longitudinal or mixed-method approaches. Third, the COVID-19 pandemic may have influenced providers’ views on integrated care by adding stress, changing team dynamics, or shifting policy priorities. However, the main motivators across all levels remain structural and policy-driven, which may have helped reduce the impact of the pandemic. Further research is needed to understand how these structural factors are organized and how they relate to one another. It is also important to explore the mechanisms through which they influence the development of integrated care.

## Conclusions

 This study explored motivators for FPTMs in urban community settings within integrated care, examining the interrelationships. It uncovered deeper-level motivators and identified key elements influencing FPTMs. The findings highlight the importance of aligning governmental policies, provider incentives, and patient engagement strategies, clarifying stakeholder roles to harmonize interests and objectives. These insights have implications for countries employing collaborative team-based primary care models.

 To effectively promote integrated care, leveraging governmental leadership in setting clear guidelines and integrating primary care with public health information systems is vital. Enhancing interdisciplinary team collaboration, performance evaluations and incentive structures can boost FPTMs’ intrinsic motivation.

 The study also found residents to be relatively passive within the incentive framework. Changing their health perceptions and behaviors through targeted guidance is crucial. Actively encouraging resident participation in health management supports collaborative integrated care, particularly benefiting low- and middle-income countries by advancing integrated care without significant new investments.

## Acknowledgements

 The authors would like to thank the FPTMs for participating in this study.

## Ethical issues

 Ethics approval for this study was granted by the Medical Ethics Committee of Tongji Medical College, Huazhong University of Science and Technology (Protocol number: [2022](S092)).

## Conflicts of interest

 Authors declare that they have no conflicts of interest.
